# Dietary diversity associated with risk of cardiovascular diseases among community-dwelling older people: A national health examination survey from Thailand

**DOI:** 10.3389/fnut.2022.1002066

**Published:** 2022-09-08

**Authors:** Chalobol Chalermsri, Shirin Ziaei, Eva-Charlotte Ekström, Weerasak Muangpaisan, Wichai Aekplakorn, Warapone Satheannopakao, Syed Moshfiqur Rahman

**Affiliations:** ^1^Department of Women's and Children's Health, Uppsala University, Uppsala, Sweden; ^2^Department of Preventive and Social Medicine, Faculty of Medicine Siriraj Hospital, Mahidol University, Bangkok, Thailand; ^3^Department of Community Medicine, Faculty of Medicine Ramathibodi Hospital, Mahidol University, Bangkok, Thailand; ^4^Department of Nutrition, Faculty of Public Health, Mahidol University, Bangkok, Thailand

**Keywords:** dietary diversity, cardiovascular diseases, cardiometabolic risk factor, older people, Thailand

## Abstract

**Background:**

Cardiovascular diseases (CVD) are the common comorbidities in older people. Healthy diet is an essential strategy to alleviate the risk of developing CVD. Dietary diversity (DD) is an indicator of diet quality. Currently, limited research exists regarding DD and CVD in older people in developing countries, such as Thailand, despite rapid growth of older population. Therefore, this study aims to determine associations of DD with the risk of CVD and the cardiometabolic risk factors among Thai older people.

**Methods:**

This cross-sectional study used the sub-sample of the fifth Thai National Health Examination Survey conducted from 2013 to 2015. A total of 6,956 older people aged 60 years and older and no pre-existing CVD were included.

Dietary diversity score (DDS) was assessed the consumption of eight food groups using food frequency questionnaires. Each food group was scored from 0 to 4. The DDS was calculated as the sum of the scores (0–32). The risk of CVD was calculated by using a Thai cardiovascular (CV) risk score. The cardiometabolic risk factors included hypertension, diabetes mellitus (DM), total cholesterol (TC), low-density lipoprotein cholesterol (LDL-C), high-density lipoprotein cholesterol (HDL-C), and triglyceride (TG) levels. Data were adjusted for a complex survey design and analysed using linear and logistic regression models.

**Results:**

In the adjusted model, DDS had a significant negative association with log-Thai CV risk score, with adjusted *β* (95% CI) values of −0.01 (−0.01, −0.01). Regarding the cardiometabolic risk factors, DDS had a significant negative association with hypertension, DM and log-TG levels, with adjusted OR (95% CI) values of 0.97 (95% CI 0.97, 0.98) for hypertension, 0.94 (0.93, 0.95) for DM, and adjusted *β* (95% CI) values of −0.002 (−0.004, −0.001) for log-TG level. DDS was positively associated with TC and LDL-C, with adjusted *β* (95% CI) values of 0.59 (0.38, 0.80) for TC and 0.59 (0.38, 0.79) for LDL-C levels, while DDS was not associated with HDL-C level.

**Conclusion:**

Higher DD was associated with a lower risk of CVD among Thai older people. The nutritional policies or interventions should encourage a diverse food intake for the prevention of CVD in this population.

## Introduction

Globally, the ageing population is continuing to increase. According to the World Health Organisation (WHO), the number of older people aged 60 years and above will increase significantly, from 12% in 2015 to 22% by 2050 (1). Due to biological degenerative processes and socioeconomic constraints, older people face greater risks of adverse health outcomes compared with younger populations (2). Cardiovascular diseases (CVD) accounted for the majority of comorbidities and are the leading cause of death in older people (3). Cardiometabolic risk factors, including hypertension, diabetes mellitus (DM) and dyslipidaemia, are influencing determinants for developing CVD (4). The degenerative changes due to the ageing process are one of the main causes of CVD too (5). In addition, inappropriate dietary intake, poor physical activity, smoking and excess alcohol consumption are common risk factors linked to these health conditions (6). Promoting a healthy lifestyle, such as healthy dietary practise and increased physical activity, is an essential strategy to control cardiometabolic risk factors and alleviate the risk of developing CVD (7, 8).

Dietary diversity (DD) is defined as the variety of food groups consumed over a reference period (9). DD is a convenient and affordable tool for assessing the variety of nutrient intake in larger population groups, particularly older people (10, 11). Although knowledge about DD and its adverse health outcomes is growing, there are still limitations. Previous studies have examined the relationship between DD and CVD; however, the results are still inconclusive (12). Although many previous studies have found the negative associations between DDS and the risk of CVD, diabetes mellitus (DM), and abnormal lipid profile (13–16), other studies could not show the significant associations (17). Furthermore, the majority of these studies have primarily focused on the middle-aged population rather than older people (12). Depending on the ageing process, the results in older people may differ from those in the middle-aged population. Additionally, the majority of past research on this topic have been conducted in high-income countries (HIC). Many socioeconomic factors have been found to be associated with DD and CVD, such as the equality of education and the health system, varying between HIC and lower- and middle-income countries (LMIC) (18). Therefore, this study focused on exploring the association between DD and CVD among older people in LMIC, adding to the body of knowledge on this topic.

Thailand is classified as an upper-middle-income country in the Southeast Asia region. By 2035, the older population in Thailand is estimated to reach 20 million, equal to more than one-third of the overall population (19). CVD, including coronary heart disease and cerebrovascular disease, are the leading causes of death in Thai older people (20). Identification of at-risk persons and promoting a healthy lifestyle, such as healthy eating, are essential strategies for primary prevention of CVD (21). To the best of our knowledge, there is no study determining the association between DD and the risk of CVD among Thai older people. Therefore, the primary objective of this study was to determine the association between DD and the risk of CVD in Thai older people. The secondary objective was to examine the association between DD and cardiometabolic risk factors, in terms of hypertension, DM and lipid profiles in Thai older people.

## Materials and methods

### Study design, setting and population

**Figure 1:**
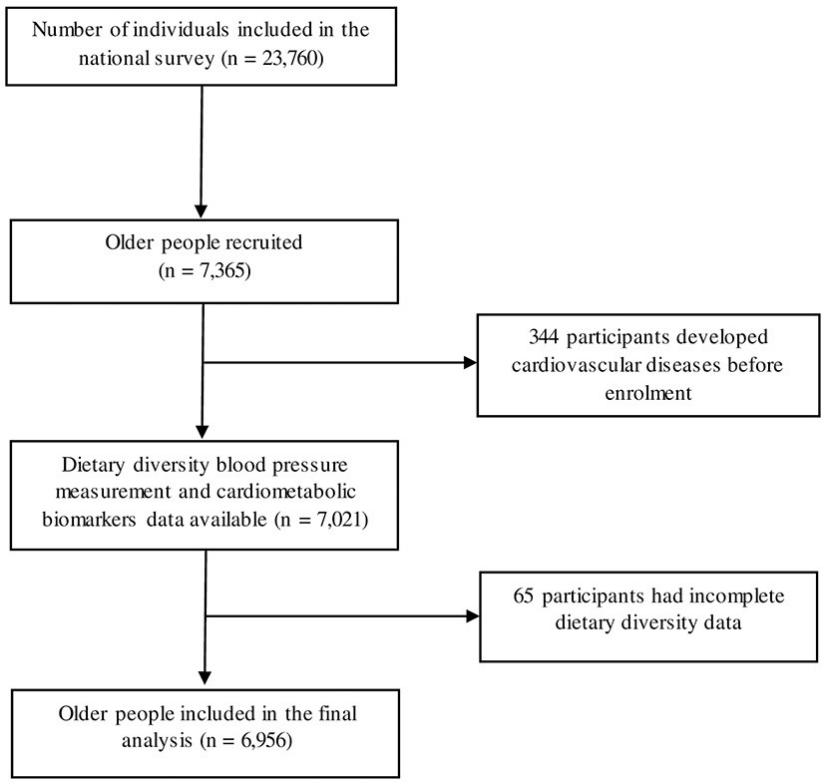
Flow diagram of study participants included in the analysis.

This study was a cross-sectional study of the fifth Thai National Health Examination Survey (NHES-V). NHES-V was a national survey conducted from 2013 to 2015 using a multistage, stratified sampling of the Thai population. The details of this survey have been previously described elsewhere (22). The inclusion criterion was age 60 years old or older. Older participants with pre-existing coronary artery disease or cerebrovascular disease, based on their history and physical examination, were excluded. A total of 7,365 older persons were recruited in this survey. Three hundred and forty-four older participants were excluded because of pre-existing CVD, such as symptomatic coronary heart disease and symptomatic cerebrovascular disease. Sixty-five older participants were excluded from the analysis because of incomplete dietary data. Thus, 6,956 older participants remained in the final analysis (Figure 1).

### Data collection

#### Dietary diversity score

Individual-level DD was determined using a 34-item semi-quantitative food frequency questionnaire (FFQ) that represented participants' dietary intake during the 30 days prior to the survey. The FFQ was administered by trained interviewers. Food images were used to encourage participants' recall and response. If participants experienced physical or communication difficulties as a result of physical or cognitive impairment, data were collected *via* their caregivers. Dietary diversity score (DDS) was calculated by using an adapted combination of DDS established by the Food and Agriculture Organisation of the United Nations (FAO) (23) and the main food groups in the food-based dietary guideline (FBDG) in Thailand (24). Although the dietary guidelines recommended a small amount of fats and oils, it is still an essential food group, having health benefits such as helping the body to absorb fat-soluble vitamins (25). Therefore, we included fats and oils in the DDS for this study. The details of DDS development have been described elsewhere (1). In summary, the DDS included eight food groups, namely (1) grains; (2) pulse, beans, nuts and seeds; (3) dairy products; (4) meat, poultry or fish; (5) eggs; (6) vegetables; (7) fruits, and (8) fats and oils. Each food group was measured on a five-point scale, depending on the frequency of consumption: never eat or eat less than once per month (0 point), eat once to three times per month (1 point), eat once to three times per week (2 points), eat 4–6 times per week (3 points) and eat once or more per day (4 points). The DDS was calculated as the sum of the score obtained from each food group. DDS ranged from 0 to 32. The higher the DDS score, the more diverse the diet. DDS were calculated based on both the number of food group and the frequency of each food group consumption. The frequency of consumption is a proxy of diet quantity, and it will improve model fit due to the higher detail of information. The previous paper in Japan compared the performance of the different scoring systems of DDS in older people. It has showed that DDS which combined the frequency of each food group consumption, and the number of food groups has higher performance to diagnose malnutrition than the scoring system which used only the number of food group (26).

#### Risk of cardiovascular diseases and cardiometabolic risk factors

The risk of CVD was calculated by using the Thai CV risk score, which was constructed based on the population cohort in Thailand (27). The Thai CV risk score included sex, age, systolic blood pressure (SBP), the diagnosis of DM, current smoking status and total cholesterol (TC) level, while diastolic blood pressure (DBP), fasting plasma glucose (FPG), Low-density lipoprotein cholesterol (LDL-C), High-density lipoprotein cholesterol (HDL-C), and triglycerides (TG) levels were not included in the score (28). This score was validated in the Thai population and used as the predictor of the percentage that would develop coronary artery disease, as well as fatal and non-fatal cerebrovascular disease in the next 10 years (27, 29). Thus, a higher score meant a higher risk of CVD.

Cardiometabolic risk factors, including hypertension, DM and lipid profiles, were collected through face-to-face interviews, blood pressure (BP) measurement and standard laboratory investigations. To get an accurate reading of the BP, participants were asked to rest in a sitting position for at least 5 min prior to the BP measurement. SBP and DBP were recorded using standard procedures by using an automatic BP monitoring device (Microlife model A100, Microlife AG, Switzerland). Each participant had three measurements of BP recorded at 1-min intervals. The first result was discarded, while the mean BP between the second and third results was used for the analysis. Hypertension was defined by the Eighth Joint National Committee (JNC 8) hypertension guidelines as SBP ≥140 mmHg or DBP ≥90 mmHg (30), and/or self-reported use of antihypertensive medications within 2 weeks.

Venous blood samples were obtained from participants after overnight fasting for 12 h to measure the FPG and lipid profiles. Blood samples were frozen and transferred to a central laboratory at the Ramathibodi Hospital, Mahidol University, Bangkok, Thailand. FPG was measured using a hexokinase enzymatic method. DM was defined by the WHO's definition as FPG ≥7.0 mmol/L (126 mg/dl) (31), and/or use of hypoglycaemic medication within 2 weeks. Serum TC and TG were analysed using enzymatic colorimetric methods. LDL-C was calculated based on the Friedewald formula for subjects with TG <4.5 mmol/L (400 mg/dl) (32) and was directly measured by the enzymatic method for those having TG ≥ 4.5 mmol/L (400 mg/dl). HDL-C was measured using homogeneous enzymatic colorimetric method. All lipid measurements were carried out using a Hitachi 917 biochemistry analyser (Roche Diagnostics, Switzerland).

#### Covariates

The following covariates were obtained during the interview: age, sex, education level, socioeconomic status (SES), place of residence, current smoking status, and current alcohol consumption. The educational level was categorised as no formal education, primary education, and secondary education and above. SES was quantified by using a wealth index, which has been described elsewhere (22). The wealth index was categorised into quintiles. The lowest quintile of the wealth index corresponded to the poorest group, and the highest corresponded to the richest group (22). The place of residence was divided into urban and rural areas. Current smoking status and current alcohol consumption were defined as smoke and alcohol consumption within 12 months before the survey date, respectively. The participant's body weight was measured in (kg) using a digital weighing scale (TANITA model HD316, Tanita Corporation, Japan). The height was measured in (cm.) using a standard metal tape. The body mass index (BMI, kg/m^2^) was calculated using the participants' weight and height during the survey. BMI was categorised based on the Asia-Pacific cut-off values: BMI <18.5 kg/m^2^ as underweight, 18.5–22.9 kg/m^2^ as normal weight, 23.0–24.9 kg/m^2^ as overweight, and ≥25.0 kg/m^2^ as obese (33).

### Data analyses

Statistical analyses were carried out using Stata17.0 (StataCorp LP, College Station, TX, USA). The analyses were adjusted for a complex survey design, which considered clustering and weighting of the data (34). This study was weighted based on the sampling probability against the 2014 registered Thai population. Descriptive statistics were presented as frequency and percentage (%) for categorical variables. The mean with standard deviation (SD) was presented for continuous variables with normal and abnormal distribution, while the median with range or interquartile range (IQR) was used for continuous variables with abnormal distribution. The association between DDS and continuous outcomes, including the Thai CV risk score and lipid profiles, was analysed using simple and multiple linear regression models and represented by beta co-efficients (*β*) and 95% CIs. The association between DDS and categorical outcomes, such as hypertension and DM, was evaluated using simple and multiple logistic regression models and represented by Odds ratio (OR) and 95% CI. The assumptions of linear regression were checked. The linearity was checked by using a scatter plot. The distribution of residuals was assessed visually using a histogram. The Thai CV risk score and TG level were log_10_-transformed to achieve an approximate normal distribution of residual for linear regression models. The homoscedasticity was also checked by using a scatter plot, and there was no heteroscedasticity. The potential confounders showing a *P*-value <0.2 in unadjusted models were entered in the adjusted model. Collinearity between categorical independent variables was checked with the chi-square test. When there was a substantial dependence between the two categorical variables, Goodman–gamma Kruskal's (G-K gamma) was used to determine the strength of the association. Sex was strongly associated with current smoking status and current alcohol consumption (*P*-value <0.05, G-K gamma −0.87 for smoking and −0.87 for alcohol consumption). Thus, these variables were not included in the final model. The generalised variance inflation factor (GVIF) with the R programme statistical software (35, 36) was also used to evaluate collinearity. The GVIF did not exceed 1.09 for any potential confounders. Therefore, there was no evidence of collinearity. The results were considered statistically significant at a level of *P*-value <0.05. Missing data accounted for 17.8% of the wealth index, while other variables such as education level accounted for <0.4% of the data. Participants with a missing wealth index were more likely to live in the urban area, abstain from alcohol consumption, have hypertension, as well as have a higher BMI and higher TC level (*P*-value <0.05). Therefore, a separate wealth index category of participants with missing wealth index data was included in all primary analyses. This method retained participants in the analysis and included their other features in the results (37). The sensitivity analyses were performed to test the robustness of the results by excluding fats and oils from the DDS.

## Results

Median age of the participants was 68.0 years (range 60.0–99.0). The median DDS was 6.0 (IQR 2.0), and the median 10-year Thai CV risk score was 21.9% (IQR 24.5). Around 55% of the participants were female. More than 90% of the participants had primary education or higher. The proportion of people with hypertension was 38.6%. Almost 12% of the older participants had DM. In the aspect of BMI, ~10% of the participants were underweight (BMI <18.5 kg/m^2^), and 54.1% of the participants were overweight and obese (BMI 23.0 kg/m^2^ and higher). The other characteristics of the participants are presented in Table 1.

**Table 1:** Characteristics of the study populations.

**Characteristics**	**Unweighted frequency**	**Weighted frequency**	**Weighted % or mean** **±SD or median (IQR)**
Age (years, mean ± SD)	6,956	6,953	69.6 ± 7.6
**Sex; %**			
Male	3,010	3,010	44.5
Female	3,946	3,943	55.5
**Education level; %**			
No formal education	655	654	9.4
Primary education	5,247	5,245	79.2
Secondary education and above	1,029	1,135	11.3
**Wealth index quintile; %**			
Quintile 1 (poorest)	1,381	1,381	29.3
Quintile 2	879	879	17.4
Quintile 3	1,015	1,014	18.1
Quintile 4	1,137	1,136	18.7
Quintile 5 (richest)	1,306	1,306	16.5
**Place of residence; %**			
Urban	3,337	3,337	40.6
**Current smoking; %**			
Yes	1,028	1,028	15.8
**Current alcohol consumption; %**			
Yes	1,417	1,417	21.0
BMI (kg/m^2^,mean ± SD)	6,861	6,858	23.7 ± 4.2
**Hypertension; %**			
Yes	2,774	2,774	38.6
**Diabetes mellitus; %**			
Yes	882	881	11.9
TC (mg/dl, mean ± SD)	6,721	6,720	200.8 ± 47.5
LDL-C (mg/dl, mean ± SD)	6,724	6,723	129.4 ± 40.1
HDL-C (mg/dl, mean ± SD)	6,715	6,714	47.6 ± 14.1
TG [(mg/dl, median (IQR)]	6,725	6,724	149.8 (85.1)

95% CI, 95% confidence interval; SD, standard deviation; IQR, interquartile range; BMI, body mass index; TC, total cholesterol; LDL-C, low-density lipoprotein cholesterol; HDL-C, high-density lipoprotein cholesterol; TG, triglyceride.

The percentage of the frequency of the consumption of each food groups in DDS is presented in the Supplementary Table 1. Almost 95% of participants consumed grains once or more per day, while <2% of them consumed fats and oils once or more per day.

### The association between DDS and Thai CV risk score

The simple and multiple linear regression analyses showed the inverse association between DDS and Thai CV risk score among older participants. After adjusting for the education level and the wealth index, DDS had a significant positive association with log-Thai CV risk score with adjusted *β* (95% CI) −0.01 (−0.01, −0.01) (Table 2).

**Table 2:** Linear regression analyses for the association between each score of DDS and log-Thai CV risk score among older participants (*n* = 6,662).

	**Thai CV risk score** ^**a**^					
	**Crude model**			**Adjusted model** ^**b**^		
	β	**95% CI**	***P*-value**	β	**95% CI**	***P*-value**
DDS	−0.01	−0.01, −0.01	<0.001	−0.01	−0.01, −0.01	<0.001

a Log-transformed.

b Adjusted for educational level and wealth index. The sample size was 6,639 due to the missing data.

DDS, dietary diversity score; Thai CV risk score, Thai cardiovascular risk score; β (95% CI), beta co-efficients and 95% confidence interval.

### The association between DDS and the prevalence of hypertension and DM

Table 3 demonstrates the simple and multiple logistic regression analyses for the association between each score of DDS and the diagnosis of hypertension and DM. DDS had a significant negative association with both hypertension and DM. In the adjusted models, the increase in each score of DDS was associated with a 3% lower risk of hypertension, with adjusted OR 0.97 (95% CI 0.97, 0.98). In addition, the increase in each score of DDS was associated with a 6% lower risk of DM, with adjusted OR 0.94 (95% CI 0.93, 0.95).

**Table 3:** Logistic regression analyses for the association between each score of DDS and cardiometabolic risk factors among older participants.

	**Crude model**				**Adjusted model**			
	**Sample size**	**OR**	**95% CI**	***P*-value**	**Sample size**	**OR**	**95% CI**	***P*-value**
**Hypertension**	6,826				6,801			
No		Reference				Reference		
Yes		0.98	9,97, 0.98	<0.001		0.97^a^	0.97, 0.98	<0.001
**Diabetes mellitus**								
No		Reference				Reference		
Yes		0.95	0.95, 0.96	<0.001		0.94^b^	0.93, 0.95	<0.001

a Adjusted for sex, age, educational level, wealth index, and place of residence.

b Adjusted for sex, age, wealth index, and place of residence.

DDS, dietary diversity score; OR (95% CI), odds ratio and 95% confidence interval.

### The association between DDS and lipid profiles

Table 4 shows the simple and multiple linear regression analyses for the association between DDS and lipid profiles. In the adjusted model, DDS had a significant positive association with TC, and LDL-C levels with adjusted *β* 0.59 (95% CI: 0.38, 0.80) for TC, adjusted *β* 0.59 (95% CI 0.38, 0.79) for LDL-C level. On the other hand, in the adjusted model, DDS had a significant negative association with log-TG, with adjusted *β* −0.002 (95% CI −0.001, −0.004). However, there was no association between DDS and HDL level in both univariate and multivariate models.

**Table 4:** Linear regression analyses for the association between DDS and lipid profiles among older participants.

**Lipid profiles**	**Crude model**				**Adjusted model**			
	**Sample size**	***β***	**95% CI**	***P*-value**	**Sample size**	***β***	**95% CI**	***P*-value**
TC	6,720	0.66	0.45, 0.87	<0.001	6,697	0.59^a^	0.38, 0.80	<0.001
LDL-C	6,723	0.62	0.42, 0.82	<0.001	6,700	0.59^a^	0.38, 0.79	<0.001
HDL-C	6,714	0.05	−0.02, 0.11	0.130	6,691	0.00^b^	−0.06, 0.06	0.985
TG^c^	6,724	−0.002	−0.004, −0.001	<0.001	6,701	−0.002^a^	−0.004, −0.001	0.008

a Adjusted for age, sex, educational level, wealth index, and place of residence.

b Adjusted for sex, educational level, wealth index, and place of residence.

c Log-transformed.

DDS, dietary diversity score; β (95% CI), beta co-efficients and 95% confidence interval; TC, total cholesterol; LDL-C, low-density lipoprotein cholesterol; HDL-C, high-density lipoprotein cholesterol; TG, triglyceride.

The robustness of the results was examined by excluding fats and oils from the DDS in sensitivity analyses. The sensitivity analyses showed similar results compared to the primary analyses.

## Discussion

This population-based study among Thai older people indicated that DDS had a negative association with the 10-year risk of CVD. Regarding the cardiometabolic risk factors, DDS also had a negative association with the prevalence of hypertension, DM and TG levels, whereas a positive association was indicated with TC and LDL-C levels.

In this study, DDS was found to be a protective factor against developing CVD among older people. This finding was consistent with a previous study conducted among Italian older people. It shows that the increased DDS operates as a protective factor against coronary heart disease (13). Furthermore, another case-control study in Korean older patients confirms that the average DDS in cerebrovascular disease patients was significantly lower than in the control group (38). However, the result remained inconsistent. A cohort study in the US examined the association between three DDS measurement scores and the risk of ischemic heart disease. There was no association between DDS and ischemic heart disease when using the scores, which simply counted the number of food groups people consumed (39). However, the outcomes of this study included fatal myocardial infarction and non-fatal coronary disease, for which the information was self-reported from the participants themselves or their next of kin, and the vital registration. The accuracy of self-reported information might be less for diagnosis of myocardial infraction (40). Moreover, the difference in the participants' characteristics, lifestyle and dietary pattern might have affected the finding.

Because several risks factors contribute to the development of CVD, the multifactorial CV risk score provided a more comprehensive assessment of CVD than single risk factors (41). In this study, DDS had a negative association with the risk of CVD, hypertension, DM and TG level, whereas it had a positive association with TC and LDL-C levels. It demonstrated that the effect of DDS in decreasing the CVD risk through the pathway of hypertension, DM and TG level outweighed the increased CVD risk due to LDL-C.

Besides the risk of cardiovascular diseases, DDS also had a negative association with the diagnosis of hypertension. In line with our finding, there are previous studies in different settings that examine the negative relationship between DD and hypertension (42, 43). However, this association is inconsistent too. A recent study in Iran could not find such an association (44). However, there are several differences in terms of baseline characteristics and the measurement tool between the study in Iran and the current study. In the cited study, around 10% of the participants having a history of ischemic heart disease still remained in the analyses. Moreover, the prevalence of smoking and BMI in the cited study is higher than in the current study. With regard to DDS, the DDS in the cited study consists of five food groups including bread-grains, vegetables, fruits, dairy, and meat, whereas beans, eggs, and fats and oils were added as main food groups of DDS in our study. The different food groups might affect the results. Consumption of beans and eggs is negatively associated with blood pressure and the prevalence of hypertension (45, 46). Therefore, the characteristics and the component of DDS might modify the results.

Additionally, DDS demonstrated a negative association with the diagnosis of DM. These findings agreed with the findings of prior studies (15, 47). After a 10-year follow-up period, a previous population-based cohort study in the UK discovered that having a high DDS was related to a decreased chance of getting type 2 DM (15). Another prospective cohort study showed the interaction between DDS and cognitive function towards the prevalence of DM among older people in Taiwan. Older people with low DDS and cognitive impairment have a higher risk of being diagnosed with DM than those with high DDS and normal cognition (47). Evidence has shown that having a wide variety of foods has a positive effect on the diagnosis of DM (15).

In this study, DDS also had a negative association with the TG level. This association is consistent with the findings of previous studies in different settings (16, 48, 49). A study among older people residing in a rehabilitation centre found a negative association between DDS and TG levels (48). This association is also observed in people with at risk of diabetes or pre-diabetes (16, 49). However, the prior study conducted among Han and Tibetan older highlanders in China reveals a different conclusion. DDS shows a positive association with TG levels in Tibetan older people but not in Han older people (50). However, Tibetan highlanders have a unique dietary pattern that is distinguishable from mainland dwellers due to tradition and environment. Tibetan highlanders eat high amounts of meat and soybean, while they eat low amounts of fish and other aquatic products, fruits and vegetables (51). As a result, the finding might have been influenced by this dietary pattern.

This study found a positive association between the DDS with TC and LDL-C levels. Previous studies examined the association between DDS and these lipid profiles, showing the inconsistent findings. A previous study conducted among Japanese older people showed that older people with greater DDS have a higher prevalence of self-reported hyperlipidaemia (10), whereas another study conducted among frail older people in the US found no significant associations between DDS and TC or LDL-C levels (48). It is difficult to explain this finding. However, a noticeable characteristic of DDS in the US study was that whole foods were counted into the food variety score, not divided into separate food groups. Mixed dishes containing multiple main ingredients may contribute to the different results when compared with this current study. Additionally, the difference in food groups for the DDS and covariates, adjusted in the model, might affect the results. In the current study, fats and oils were included in DDS regardless of the type of fat. Moreover, the association between DDS and TC and LDL-C might be affected by fats and oils.

In this study, no association was examined between DDS and HDL-C level. Consistent with our findings, the previous studies in older Chinese people failed to discover the association between DDS and HDL-C level. In terms of the association between DDS and HDL-C level (50), it shows that there are several factors that affect this association, such as physical activities.

Several hypotheses may explain these findings. The first hypothesis involves biological processes. DDS has a relationship with gut microbiota. Although the gut microbiota may change as a result of ageing or chronic disease, the dietary component remains a critical determinant of the gut microbiota (52). High variety of food consumption has been found to be associated with a more diverse and healthier gut microbiota pattern (53). A recent review states the interaction between gut microbiota and coronary artery diseases (54). Additionally, the gut microbiota has been identified as a critical factor in the development of DM by affecting glucose metabolism, insulin resistance in multiple organs and inflammatory process (55). Thus, the diversity of food consumption might foster a healthy pattern of gut microbiota and reduce the chances of acquiring DM. The second hypothesis could be explained by the health consciousness behaviour. Older people have higher level of health consciousness behaviours, such as having a healthy lifestyle and attending health examination, compared with younger people (56). The previous qualitative study in Thai older people has shown that older people and their caregivers recognised the importance of food towards older people's health, and older people tend to follow the healthcare professional's recommendations. Thus, older people might adapt their lifestyle towards the healthier lifestyle than the younger population (57).

In Thailand, the concern about older people has been included in the twelfth nation economic and social development plan of Thailand. Currently, there are several policies aimed to enhance older people's health and wellbeing such as the national plan for older person (58). These policies focus on health promotion and diseases prevention among older people. In addition, the life-long education is encouraged in this population. Eating the variety of food is a component in Thai healthy eating index which is a tool to evaluate dietary quality and monitor the change of eating practise. Moreover, this index can be used as the tool for the nutritional educational program and health promotion program in the combination with the food based dietary guideline (24, 59).

### Strengths and limitations

Strengths of this study included the use of data from a large cohort sample size of the older Thai population. Currently, knowledge about DD and CVD among older people in LMIC is still limited; therefore, the results from this study population of Thai older people can add additional comprehensive knowledge and help to reduce this gap. Moreover, this study used Thai CV risk score, which was the validated tool for estimating risk of CVD in Thailand. Ethnicity is a significant determinant of CVD. The screening tool for CVD in western and Asian countries should be established separately (60). Thus, the Thai CV risk score used in this study was a suitable tool for assessing the risk of CVD in the older Thai population. Additionally, this study had a high degree of data reliability since the dietary diversity data were collected on a personal level from the individuals themselves or their caregivers. To assist participants if they suffered memory loss in relation to their dietary intake, interviewers used photographs of food. Furthermore, older people were interviewed regarding their food consumption habits, which may reflect an individual's exact food intake rather than a snapshot in time. Finally, the analyses included a distinct category of participants with missing wealth index data. This method retained a greater number of participants in multivariate models than the complete case analysis.

However, it should be noted that the current study had limitations. Due to the cross-sectional nature of this investigation, temporal causation could not be established. In addition, the DDS was calculated using a semi-quantitative FFQ. As a result, it may not accurately display the amount of each food item consumed or food group. Moreover, DDS aimed to assess the variety of food group consumption. Thus, fats and oils were included in the DDS. It might not totally differentiate between healthy and unhealthy diet as well. Similar to other CV risk scores, Thai CV risk score did not validate for the extremely old age (61). Older participants might have a high Thai CV risk score due to their age alone. Moreover, the CV risk score accounted for only main CVD risk factors and not all risk factors. Another limitation was that many participants had missing data, especially on SES, and the missing pattern was not random. This might affect the results; however, we included a separate wealth index category of participants with missing wealth index data in order to retain these participants in the analyses. Finally, although we endeavoured to account for the potential confounders, a number of residual confounders, such as physical activities, the health system and the effect of caregivers, still persisted.

### Recommendations

This study examined the negative association between DDS and the risk of CVD and the cardiometabolic risk factor among older Thai people. CVD is accounted as the most common NCD among older population. To prevent NCD among older people, the variety of food should be encouraged through the nutritional educational program (59). Furthermore, DD can be used as the tool for evaluating the lifestyle intervention strategies among older people with CVD or cardiometabolic risk factors.

## Conclusions

This study demonstrated a negative association between DDS and risk factors of CVD. Thus, in this setting of older Thai people, increases in dietary diversity may have a favourable effect on their risk of developing metabolic syndrome. Thus, this highlights the importance of nutritional interventions and strategies that promote healthy eating habits, characterised by a diversified diet in order to prevent CVD in this population.

## Data availability statement

The original contributions presented in the study are included in the article/Supplementary material, further inquiries can be directed to the corresponding author/s.

## Ethics statement

The studies involving human participants were reviewed and approved by the Ethical Committee for Research in Human Subjects, Faculty of Medicine, Siriraj Hospital, Mahidol University, Thailand (COA Si 076- 2021). The patients/participants provided their written informed consent to participate in this study.

## Author contributions

CC, SZ, E-CE, SMR, WA, WS, and WM: conceptualisation and methodology and reviewing the paper. WA and WS: the data collection. CC and SMR: analysis of the data, drafting, and editing of the paper. All authors read, reviewed, and approved the final manuscript.

## Funding

This research received no specific grant from any funding agency. The fifth National Health Examination Survey of Thailand was supported by the Bureau of Policy and Strategy, Ministry of Public Health, Thai Health Promotion Foundation, National Health Security Office, Thailand, and Health System Research Institute.

## Conflict of interest

The authors declare that the research was conducted in the absence of any commercial or financial relationships that could be construed as a potential conflict of interest.

## Publisher's note

All claims expressed in this article are solely those of the authors and do not necessarily represent those of their affiliated organizations, or those of the publisher, the editors and the reviewers. Any product that may be evaluated in this article, or claim that may be made by its manufacturer, is not guaranteed or endorsed by the publisher.
