# Supplementary material for: Dietary diversity associated with risk of cardiovascular diseases among community-dwelling older people: A national health examination survey from Thailand
Source: Front Nutr. 2022 Sep 8;9:1002066. doi: 10.3389/fnut.2022.1002066 (PMC9493071; doi:10.3389/fnut.2022.1002066)
Supplement: Supplementary file 1 [file Table_1.DOCX]

Supplementary Material

**Supplementary Table 1. The proportion of the frequency of consumption of each food group in DDS (n= 6,956)**

| **Food group** | **Frequency of consumption (%)** | | | | |
| --- | --- | --- | --- | --- | --- |
|  | **never or <1 / month** | **1–3 times / month** | **1–3 times / week** | **4–6 times / week** | **>1 times / day** |
| **Grains** | 0.1 | 0.3 | 1.3 | 3.6 | 94.8 |
| **Pulse, beans, nuts and seeds** | 19.9 | 22.3 | 32.3 | 13.9 | 11.7 |
| **Dairy products** | 41.8 | 15.2 | 22.1 | 9.4 | 11.5 |
| **Meat, poultry or fish** | 0.3 | 1.8 | 19.8 | 37.6 | 40.6 |
| **Eggs** | 9.3 | 13.4 | 39.4 | 25.2 | 12.7 |
| **Vegetables** | 4.3 | 11.6 | 32.0 | 27.5 | 24.6 |
| **Fruits** | 7.1 | 17.4 | 40.9 | 18.7 | 15.9 |
| **Fats and oils** | 26.7 | 34.5 | 29.7 | 7.4 | 1.8 |
